# Analysis of the Stimulative Effect of Arginine on Translation Initiation of Protein Synthesis in Skeletal Muscle

**DOI:** 10.3390/nu17182981

**Published:** 2025-09-17

**Authors:** Daisuke Suzuki, Yuki Takami, Yusuke Sato, Yuka Toyoshima, Fumiaki Yoshizawa

**Affiliations:** 1 Department of Biological Production Science, United Graduate School of Agricultural Science, Tokyo University of Agriculture and Technology, Fuchu 183-8509, Tokyo, Japan; dadadada0616@gmail.com (D.S.); yukat@cc.utsunomiya-u.ac.jp (Y.T.); 2 Department of Agrobiology and Bioresources, School of Agriculture, Utsunomiya University, Utsunomiya 321-8505, Tochigi, Japan; y.takami612@gmail.com; 3 Department of Animal Science, School of Agriculture, Tokai University, Mashiki 861-2205, Kumamoto, Japan; sato.yusuke.k@tokai.ac.jp

**Keywords:** arginine, skeletal muscle, protein synthesis, leucine, mTORC1, 4E-BP1, S6K1

## Abstract

**Background:** Arginine (Arg) is thought to potentially stimulate protein synthesis. Although the detailed mechanism by which Arg regulates protein synthesis is not fully known, it is believed to occur primarily through the mechanistic target of rapamycin complex 1 (mTORC1)-dependent activation of translation initiation. The aim of this study was to evaluate the ability of Arg to stimulate translation initiation to upregulate protein synthesis and identify the possible signaling pathways involved in the stimulatory effect of Arg on mRNA translation in skeletal muscle. **Methods:** Overnight-fasted mice were intraperitoneally injected with Arg, sacrificed 1 h later, and then the gastrocnemius muscles were excised. In addition, to determine the mechanism by which Arg stimulates translation initiation in skeletal muscle, we used mouse-derived C2C12 myotubes. Cells were preincubated with several inhibitors of intracellular signaling or the G protein–coupled receptor, Class C, group 6, subtype A (GPRC6A) antagonist, and then added to the culture with Arg. Phosphorylation of 4E-binding protein 1 (4E-BP1) and ribosomal protein S6 kinase (S6K1) as markers of mTORC1-dependent protein synthesis activity was measured. **Results:** Intraperitoneal injection of Arg increased 4E-BP1 and S6K1 phosphorylation. In C2C12 myotubes, Arg addition significantly increased the phosphorylation of 4E-BP1 and S6K1, and this upregulation was attenuated by pretreatment with the mTORC1 inhibitor rapamycin. In addition, pretreatment with the PI3K inhibitor LY294002, the AKT inhibitor MK-2206, and the GPRC6A antagonist calindol completely inhibited Arg-upregulated 4E-BP1 and S6K1 phosphorylation. **Conclusions:** The findings of this study suggest that Arg stimulates the initiation of mRNA translation via the GPRC6A/PI3K/AKT/mTORC1 signaling pathway, thereby stimulating protein synthesis in skeletal muscle.

## 1. Introduction

Although amino acids are the “building blocks” of proteins, free amino acids not incorporated into proteins are also present in cells and blood. Research has clearly demonstrated that free amino acids act as important signaling molecules that regulate both anabolic and catabolic pathways [1,2,3]. Specifically, the mechanistic target of rapamycin complex 1 (mTORC1) signaling cascade, which regulates cell growth and metabolism, is particularly sensitive to changes in plasma levels of essential amino acids and leucine (Leu) [4]. Amino acids in general, but especially Leu, stimulate the translation initiation step of protein synthesis in skeletal muscle by phosphorylating eukaryotic initiation factor 4E-binding protein 1 (4E-BP1) and ribosomal protein S6 kinase (S6K1), two key downstream targets of the mTORC1 signaling pathway [5,6,7]. Phosphorylation of S6K1 and 4E-BP1 is associated with an acceleration of mRNA translation initiation, which in turn leads to the stimulation of protein synthesis [8]. Phosphorylation of 4E-BP1 at multiple sites induces its release from eIF4E, allowing cap-dependent translation to proceed [9,10]. By contrast, phosphorylation of S6K1 promotes protein synthesis and cell growth through multiple substrates, including ribosomal protein S6, which subsequently promotes translation initiation [10,11,12]. Leu is likely the most potent mTORC1 activator among all amino acids [5], and the mechanism by which Leu regulates mTORC1 activity has been elucidated. Leu regulates the mTORC1 pathway through Leu sensors, such as leucyl-tRNA synthetase (LRS) and sestrin1/2 [13,14].

Leu is not the only amino acid that acts as a nutrient signal to stimulate protein synthesis, however. Several notable studies have suggested that arginine (Arg), a conditionally essential amino acid, activates mTORC1 to stimulate protein synthesis, potentially via a mechanism similar to that of Leu [15,16,17]. Cytosolic arginine sensor for mTORC1 subunit 1 (CASTOR1) is an intracellular Arg sensor discovered approximately 10 years ago, which regulates the activity of the mTORC1 pathway [18]. Arg likely acts in the intact form, as its metabolism is not required for mTORC1 activation. Whether this effect is direct or indirect remains to be elucidated, however. Arg specifically stimulates the secretion of growth hormone (GH) [19], insulin [20], insulin-like growth factor 1 (IGF-1) [21], and other hormones. Based on these reports, it can be hypothesized that Arg enhances protein synthesis in muscle through IGF-1 and subsequent mTORC1 activation. Although strong evidence indicates that the IGF-1/mTORC1 pathway is a crucial regulator of muscle mass, nitric oxide (NO) was recently suggested as a common regulator of this process [22]. Arg is the only substrate for NO synthesis [23]. In C2C12 cells, Arg was shown to activate the mTORC1/S6K1 signaling pathway in a NO-dependent manner [24]. However, studies on the signaling function of Arg have predominantly utilized cell lines of non-muscle origin. It is thus unclear whether the stimulative effect of Arg on protein synthesis in muscle is due to Arg itself or its metabolite, NO.

The aim of this study was to evaluate the ability of Arg to stimulate protein synthesis in skeletal muscle and compare this with the stimulatory effect of Leu. The effects of Arg and Leu on muscle protein synthesis at the level of translation initiation were investigated in vivo in mice and in vitro in differentiated mouse C2C12 myotubes. Using C2C12 cells, we also examined the possible signaling pathways involved in translational control of muscle protein synthesis by Arg.

## 2. Materials and Methods

### 2.1. Animal Experiments

Five-week-old male ICR mice were purchased from Charles River Japan (Yokohama, Japan). The mice were housed at 22 °C under a 12-h light-dark cycle (lights on from 0700–1900 h). Mice were allowed free access to water and a 20% casein diet based on the AIN-93G for 1 week. After 18 h of fasting, the mice were administered Leu or Arg via oral gavage or intraperitoneal injection. For oral gavage, the mice were given 10.29 mmol/kg body weight (bw) of Leu (1.35 g/kg bw) or 3.08 mmol/kg bw of Arg (0.54 g/kg bw). These amounts were equivalent to the amount consumed in a 24-h period by male Sprague–Dawley rats provided free access to AIN-93G standard diet. For intraperitoneal injection, the mice were given 4.6 mmol/kg bw of Leu (0.60 g/kg bw) or Arg (0.80 g/kg bw) [25]. For both oral and intraperitoneal administration, control mice received an equivalent volume of water or saline. At 1 h after administration, the mice were sacrificed under isoflurane anesthesia (Viatris, Tokyo, Japan), and blood samples were obtained from the inferior vena cava. Subsequently, the gastrocnemius muscle was extracted from both hindlimbs. The animal care protocol was approved by the Utsunomiya University Animal Research Committee under the Guidelines for Animal Experiments of Utsunomiya University (approval code: A21-0008; approval date: 21 April 2021).

Whole blood was incubated for 2 h at room temperature to allow for clotting and then subjected to centrifugation at 2700× *g* for 10 min at 4 °C. The resulting serum was stored at −80 °C until analysis.

The extracted muscles were homogenized using a polytron homogenizer in 7 times the tissue weight of ice-cold homogenizing buffer (20 mM HEPES [pH 7.4], 100 mM KCl, 0.2 mM EDTA, 2 mM EGTA, 1 mM DTT, 50 mM NaF, 50 mM β-glycerophosphate, 0.1 mM PMSF, 1 mM benzamidine, and 0.5 mM sodium orthovanadate). The muscle homogenates were centrifuged at 10,000× *g* for 10 min at 4 °C. The supernatants were collected and diluted with an equal volume of 2× SDS buffer and heated for 3 min at 100 °C. The prepared samples were then used for immunoblotting. To evaluate the phosphorylation state of 4E-BP1, the collected supernatants were heated for 10 min at 100 °C [26], re-centrifuged at 10,000× *g* for 30 min at 4 °C, and then the supernatants were collected and mixed with an equal volume of 2× SDS buffer. The samples were heated for 3 min at 100 °C, and all samples were stored at −80 °C until immunoblotting analysis.

### 2.2. Analysis of Serum IGF-1 Level

The concentration of serum IGF-1 was determined using an IGF-1 ELISA kit (Proteintech, Rosemont, IL, USA) according to the manufacturer’s recommendations.

### 2.3. Serum Amino Acid Analysis

Serum concentrations of amino acids were determined using a UF-Amino Station system (Shimadzu, Kyoto, Japan).

### 2.4. Cell Culture

Mouse C2C12 myoblasts were purchased from the American Type Culture Collection (Manassas, VA, USA). The cells were cultured in high-glucose DMEM (Life Technologies, Carlsbad, CA, USA) supplemented with 10% fetal bovine serum (Life Technologies) and antibiotics (100 U/mL penicillin and 100 mg/mL streptomycin; Nacalai Tesque, Kyoto, Japan). The myoblasts were grown to confluence, and myogenic differentiation was induced by switching the medium to high-glucose DMEM supplemented with 2% horse serum (Life Technologies) and antibiotics. The cells were then maintained in high-glucose DMEM containing 2% horse serum and antibiotics for 4–5 days to enable differentiation into myotubes. The medium was replaced every 2 days. All cultured cells were maintained in a humidified atmosphere of 95% air and 5% CO_2_ at 37 °C.

To stimulate differentiated myotubes using various amino acids, the cells were washed twice with PBS, and the medium was changed to amino acid–free HEPES-buffered Krebs-Henseleit solution containing 0.1% bovine serum albumin, 2 mM sodium pyruvate, and 5 mM D-glucose. The cells were then starved of serum and amino acids for 4 h, after which the medium was changed to an amino acid–free medium supplemented with single or several combinations of amino acids. The concentration of each amino acid added and the time of stimulation are indicated in the figure legends.

To identify the signaling pathways involved in Arg-simulated translation initiation, myotubes were pre-treated with various inhibitors: rapamycin (10 nM, Cell Signaling Technology, Danvers, MA, USA), L-NMMA (0.2 to 2 mM, DOJINDO, Kumamoto, Japan), LY294002 (1 µM, Sigma-Aldrich, St. Louis, MO, USA), MK-2206 (0.5 µM, Selleck Chemicals, Houston, TX, USA), U0126 (0.5 µM, Cell Signaling Technology), Sch772984 (0.5 µM, Funakoshi, Tokyo, Japan), or calindol (25 µM, Sigma-Aldrich) for the time periods indicated in the figure legends. The medium was then changed to amino acid–free medium containing 5 mM Arg and each inhibitor, and the cells were incubated for 15 min.

After treatment, the cells were harvested using RIPA buffer (Nacalai Tesque) containing phosphatase inhibitor cocktail (Nacalai Tesque). The cells were sonicated on ice to prepare lysates, and samples for immunoblotting were prepared as described for the case of muscle homogenates.

### 2.5. Immunoblotting

Protein samples from gastrocnemius muscle and myotubes were separated using SDS-PAGE and transferred onto PVDF membranes (Bio-Rad Laboratories, Hercules, CA, USA). The membranes were blotted at 4 °C overnight with the following primary antibodies: anti–4E-BP1 (1:800, #sc-230, Santa Cruz Biotechnology Inc., Dallas, TX, USA), anti-S6K1 (1:1000, #9202, Cell Signaling Technology), anti–phospho-ERK1/2 (1:1000, #9101, Cell Signaling Technology), anti-ERK1/2 (1:1000, #9102, Cell Signaling Technology), anti–phospho-Ser473-AKT (1:1000, #4060, Cell Signaling Technology), anti–phospho-Thr308-AKT (1:1000, #4056, Cell Signaling Technology), anti-AKT (1:1000, #9272, Cell Signaling Technology), and anti–β-tubulin (1:1000, #86298, Cell Signaling Technology). After incubation with appropriate secondary antibodies, proteins were visualized using the ChemiDoc™ XRS+ System (Bio-Rad Laboratories) with chemiluminescence detection kit (Cytiva, Marlborough, MA, USA) according to the manufacturer’s instructions. The intensity of immunoreactive bands was quantified using Image Lab software (version 6.0.1, Bio-Rad Laboratories).

The phosphorylation states of 4E-BP1 and S6K1 were evaluated as previously described [5,6,26]. Briefly, 4E-BP1 can be categorized into three electrophoretic forms, designated α, β, and γ, depending on the phosphorylation level. The γ form is the most phosphorylated form and exhibits the slowest electrophoretic mobility. Among the three forms, γ is the only form that does not bind to eIF4E. Accordingly, phosphorylation of 4E-BP1 was evaluated as a percentage of the phosphorylation of the γ form relative to all three forms. Similarly, S6K1 can be categorized into several electrophoretic forms depending on the phosphorylation level [27]. To evaluate S6K1 phosphorylation, the ratio of the more slowly migrating hyperphosphorylated forms of S6K1 (β, γ, and δ forms of S6K1) to the total immune reactivity was quantified because it is the hyperphosphorylated forms that exhibit kinase activity.

### 2.6. Statistical Analyses

All data are expressed as the mean with standard error. The data were evaluated using analysis of variance (ANOVA) followed by the Tukey-Kramer multiple comparisons test for multiple groups to identify significant differences (*p* < 0.05). Statistical analyses were performed using Microsoft Excel software (version 2019, Microsoft Corporation, Redmond, WA, USA).

## 3. Results

### 3.1. Effects of Arg on Translation Initiation in Skeletal Muscle of Mice

**Figure 1:**
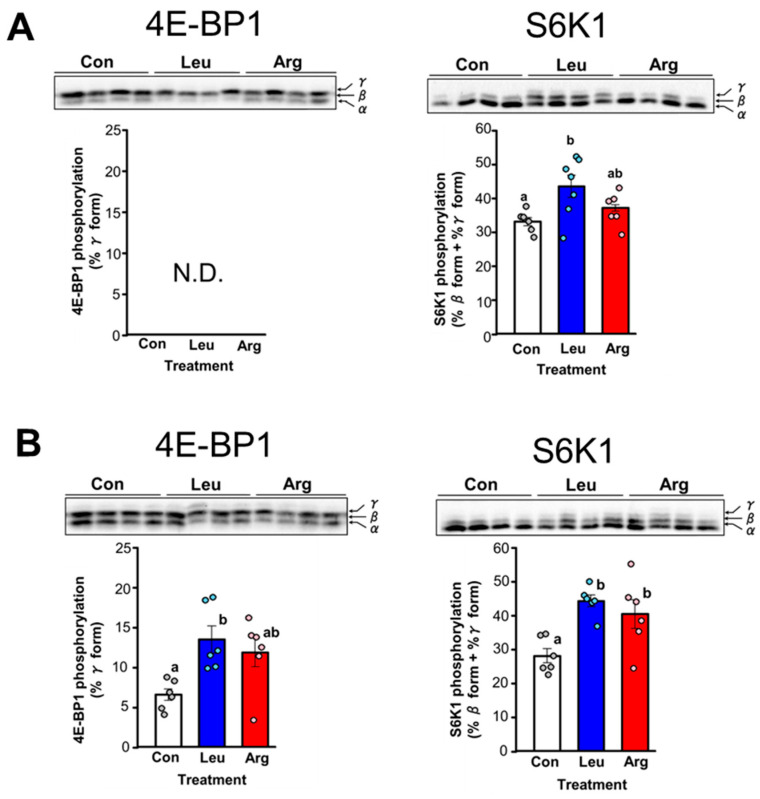
Effect of leucine (Leu) or arginine (Arg) administration on translation initiation in skeletal muscle of food-deprived mice. Mice were administered Leu or Arg either orally (**A**) or intraperitoneally (**B**), and skeletal muscle (gastrocnemius) was excised 1 h after administration. Gastrocnemius extracts were subjected to SDS-PAGE, and the phosphorylation states of 4E-binding protein 1 (4E-BP1) and ribosomal protein S6 kinase (S6K1) were determined by immunoblot analysis. 4E-BP1 resolved into three bands on SDS-polyacrylamide gels, with the top band (γ-band) corresponding to the most highly phosphorylated species. The bar graph displays the amount of 4E-BP1 in the γ-phosphorylated form, expressed as a percentage of total 4E-BP1. S6K1 resolved into multiple electrophoretic forms on SDS-polyacrylamide gels. The rapidly migrating band was arbitrarily designated α, and the more slowly migrating bands as β and γ, respectively. The bar graph displays the amount of S6K1 in the β and γ forms, expressed as a proportion of total S6K1. (**A**) Phosphorylation states of 4E-BP1 and S6K1 in skeletal muscle of food-deprived mice (Con) or 1 h after oral administration of Leu or Arg. Values are the mean ± SE for mice in the Con (*n* = 6), Leu (*n* = 7), and Arg (*n* = 6) groups. Values not sharing the same superscript letter differed significantly (*p* < 0.05) according to the Tukey-Kramer multiple comparison test. Insets: results of representative immunoblots with the positions of the α-, β-, and γ-forms of 4E-BP1 or S6K1 noted to the right. N.D. indicates not detected. (**B**) Phosphorylation states of 4E-BP1 and S6K1 in skeletal muscle of food-deprived mice (Con) or 1 h after intraperitoneal administration of Leu or Arg. Each value is the mean ± SE for mice in the Con (*n* = 6), Leu (*n* = 6), and Arg (*n* = 6) groups.

In this study, the ability of Arg to stimulate translation initiation in skeletal muscle in vivo was examined in comparison with that of Leu. Leu is known to stimulate muscle protein synthesis at the level of translation initiation via mTORC1-mediated phosphorylation of 4E-BP1 and S6K1 [5]. Therefore, the phosphorylation states of 4E-BP1 and S6K1 were examined as an indicator of translation initiation activity. Oral administration of Leu to food-deprived mice significantly increased the level of S6K1 phosphorylation, as reported previously in rats (Figure 1A). By contrast, oral administration of Arg had no observable effect on the phosphorylation state of S6K1 (Figure 1A). We also looked at the effect of oral administration of Leu or Arg on 4E-BP1 phosphorylation (Figure 1A). The most highly phosphorylated form of 4E-BP1, the γ-form, was not detected in any group (Figure 1A).

The serum concentration of Leu was 2.7-fold greater in mice orally administered Leu compared with control mice (Table 1). By contrast, oral administration of Arg resulted in a 2.0-fold increase in the serum concentration of Arg compared with control mice (Table 1). Contrary to expectations, the serum concentrations of administered amino acids did not increase as expected. The serum concentration of Arg did not increase sufficiently, which may be why the Arg effect could not be confirmed. Therefore, we attempted to further increase the serum concentration of Arg via intraperitoneal administration. Intraperitoneal injection of Arg increased the serum concentration by approximately 4.0-fold compared with the control group (Table 1), and this was accompanied by a significant increase in S6K1 phosphorylation in the muscle (Figure 1B). Phosphorylation of 4E-BP1 tended to increase in Arg-injected mice, although the change was not significant (Figure 1B). Intraperitoneal administration of Leu increased the serum Leu level by 3.9-fold compared with control mice (Table 1). Phosphorylation of both 4E-BP1 and S6K1 increased significantly in Leu-injected mice (Figure 1B). This result was consistent with previous reports showing that oral administration of Leu enhanced phosphorylation of 4E-BP1 and S6K1 in rat skeletal muscle [5,26,28,29]. These results suggest that both Arg and Leu stimulate translation initiation in skeletal muscle via activation of the mTORC1 pathway.

**Figure 2:**
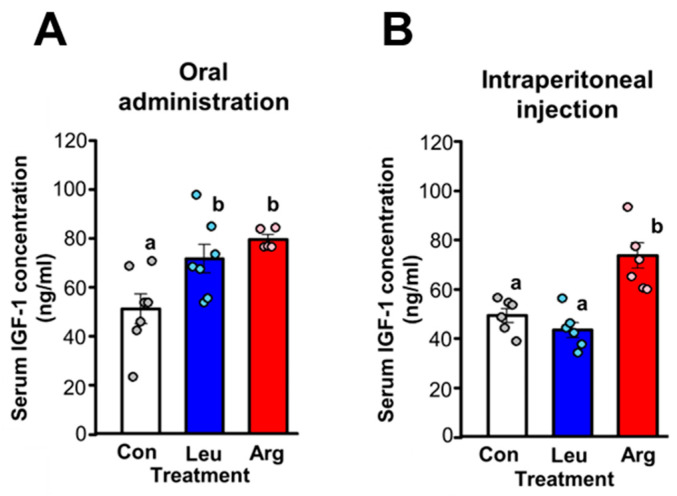
Effect of Leu or Arg administration on serum insulin-like growth factor 1 (IGF-1) level in food-deprived mice. (**A**) Serum IGF-1 concentration in food-deprived mice without Leu or Arg (Con) and food-deprived mice orally administered Leu or Arg. Each value is the mean ± SE, for mice in the Con (*n* = 7), Leu (*n* = 7), and Arg (*n* = 5) groups. Values not sharing the same superscript letter differed significantly (*p* < 0.05) according to the Tukey-Kramer multiple comparison test. (**B**) Serum IGF-1 concentration in food-deprived mice without Leu or Arg (Con) and food-deprived mice intraperitoneally administered Leu or Arg. Each value is the mean ± SE for mice in the Con (*n* = 6), Leu (*n* = 6), and Arg (*n* = 6) groups.

We also measured serum levels of IGF-1, as Arg administration reportedly increases levels of circulating IGF-1 [30], leading to enhanced translation initiation [31]. The serum IGF-1 concentration increased following either oral administration or intraperitoneal injection of Arg (Figure 2). Nevertheless, only intraperitoneal injection of Arg resulted in an increase in S6K1 phosphorylation (Figure 1).

### 3.2. Effect of Arg on Translation Initiation in C2C12 Myotubes

**Figure 3:**
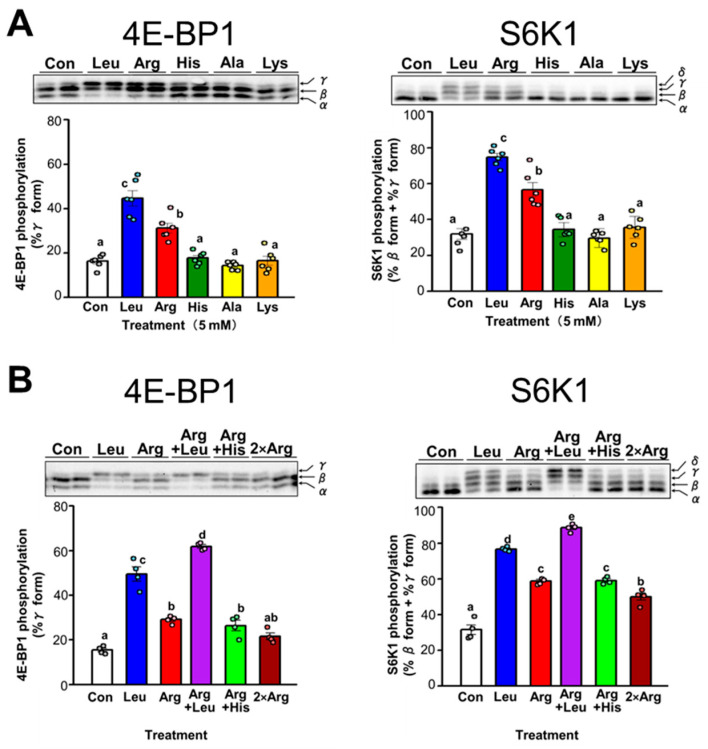
Effect of various amino acids on translation initiation in C2C12 myotubes. Differentiated C2C12 myotubes were starved of serum and amino acids for 4 h before treatment with single or combinations of amino acids. Cell lysates were prepared and subjected to SDS-PAGE, and then the phosphorylation states of 4E-BP1 and S6K1 were determined by immunoblot analysis. 4E-BP1 resolved into three bands on SDS-polyacrylamide gels, with the top band (γ-band) corresponding to the most highly phosphorylated species. The bar graph displays the amount of 4E-BP1 in the γ-phosphorylated form, expressed as a percentage of total 4E-BP1. S6K1 resolved into multiple electrophoretic forms on SDS-polyacrylamide gels. The rapidly migrating band was arbitrarily designated α, and the more slowly migrating bands as β, γ, and δ, respectively. The bar graph displays the amount of S6K1 in the β, γ, and δ forms, expressed as a proportion of total S6K1. (**A**) Cells were incubated for 30 min in amino acid–free medium supplemented with 5 mM Leu, Arg, histidine (His), alanine (Ala), or lysine (Lys). Each value is the mean ± SE; *n* = 6. Values not sharing the same superscript letter differed significantly (*p* < 0.05) according to the Tukey-Kramer multiple comparison test. (**B**) Cells were incubated in amino acid–free medium supplemented with 5 mM Leu, 5 mM Arg, 5 mM Arg + 5 mM Leu (Arg + Leu), 5 mM Arg + 5 mM His (Arg + His), or 10 mM Arg (2 × Arg) for 15 min. Each value is the mean ± SE; *n* = 4.

The effect of Arg on translation initiation in skeletal muscle was examined in detail using differentiated C2C12 myotubes as an in vitro model of skeletal muscle. The effect of administration of several amino acids, including Arg and Leu, on the phosphorylation of 4E-BP1 and S6K1 was evaluated by adding a single amino acid to C2C12 cells cultured in amino acid–free medium. Among the tested amino acids, only Arg and Leu significantly increased the phosphorylation states of 4E-BP1 and S6K1 (Figure 3A). Increased phosphorylation of 4E-BP1 or S6K1 was observed only when a specific amino acid was added, indicating that this change was not due simply to the addition of an amino acid. Subsequently, we examined the effect on translation initiation of culturing cells in the presence of both Leu and Arg. Cells were treated with Leu and Arg either individually or in combination, and the phosphorylation states of 4E-BP1 and S6K1 were compared. Simultaneous addition of both Arg and Leu to the culture medium resulted in a greater increase in phosphorylation of 4E-BP1 and S6K1 than observed when cells were treated with each amino acid alone (Figure 3B). Leu and Arg exerted an additive effect on the phosphorylation of 4E-BP1 and S6K1 (Figure 3B). By contrast, no difference was observed in the phosphorylation of 4E-BP1 and S6K1 between cells treated with only Arg and those treated with histidine and Arg (Figure 3B). Furthermore, when the amount of Arg added was doubled, no further increase in 4E-BP1 and S6K1 phosphorylation was observed; rather, the phosphorylation level decreased slightly. This suggests that the effect of Arg on S6K1 phosphorylation reaches its maximum between 1 and 2 times the amount of Arg added, specifically between 5 and 10 mM.

### 3.3. Comparison of the Effect of Arg and Leu on Translation Initiation in C2C12 Myotubes

**Figure 4:**
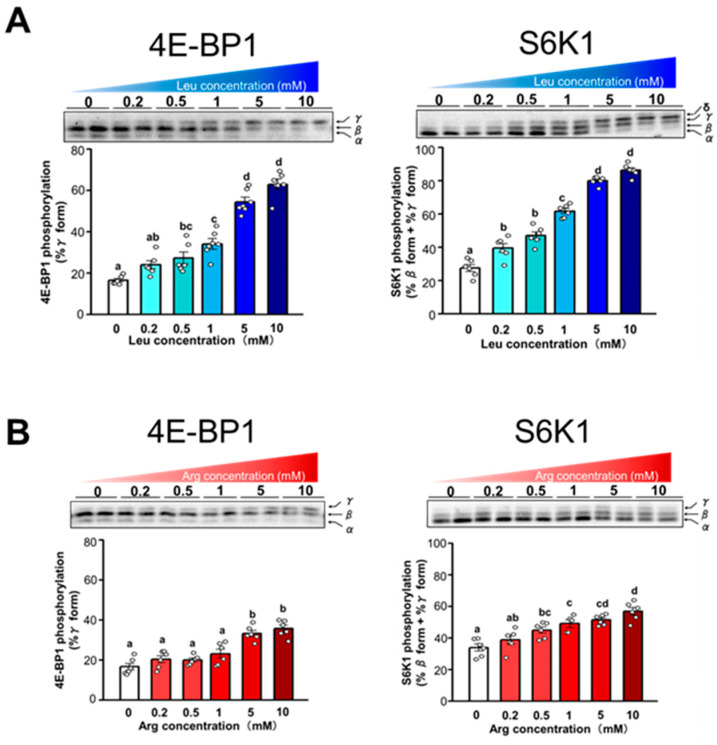

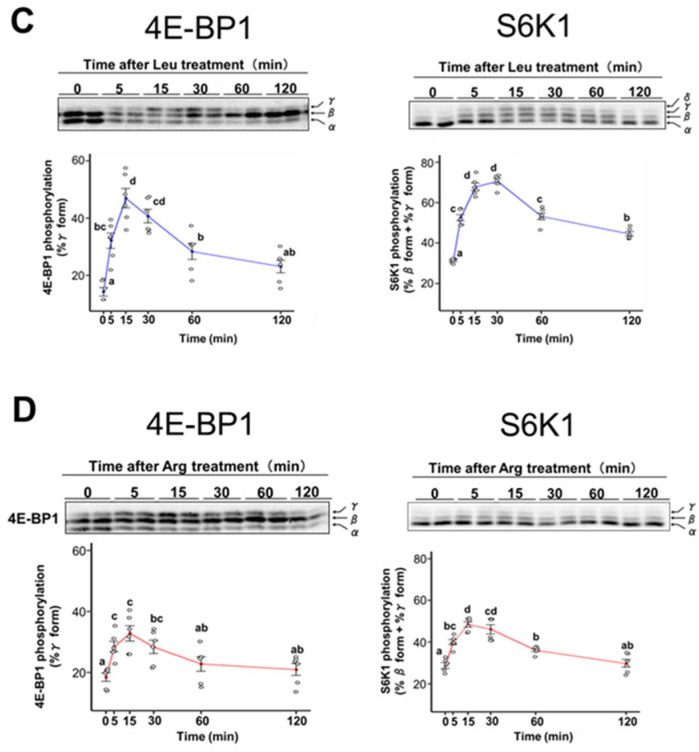

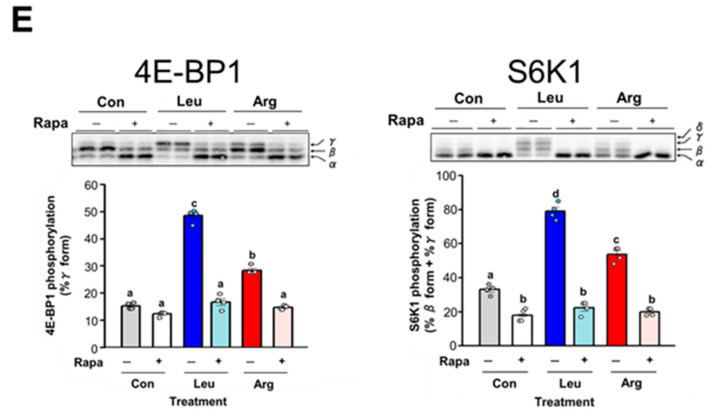
Comparison of the effect of Leu and Arg on translation initiation in C2C12 myotubes. Differentiated C2C12 myotubes were starved of serum and amino acids for 4 h and then subjected to each experiment. Cell lysates were prepared and subjected to SDS-PAGE, and then the phosphorylation states of 4E-BP1 and S6K1 were determined by immunoblot analysis. (**A**,**B**) Dose-response effect of Leu (**A**) and Arg (**B**) on the phosphorylation of 4E-BP1 and S6K1. Cells were incubated with Leu or Arg at concentrations of 0.2, 0.5, 1, 5, and 10 mM for 30 min. (**C**,**D**) Time-course effect of Leu (**C**) and Arg (**D**) on the phosphorylation of 4E-BP1 and S6K1. Cells were incubated in amino acid–free medium supplemented with 5 mM Leu or Arg for 5, 15, 30, 60, and 120 min. (**E**) Effect of the mechanistic target of rapamycin complex 1 (mTORC1) inhibitor rapamycin (Rapa) on Leu- and Arg-induced phosphorylation of 4E-BP1 and S6K1. Cells were pre-treated with or without 10 nM Rapa for 1 h and then incubated with 5 mM Leu or Arg for 15 min. Each value is the mean ± SE; *n* = 4–6. Values not sharing the same superscript letter differed significantly (*p* < 0.05) according to the Tukey-Kramer multiple comparison test.

The dose-dependent effect of Leu and Arg on the phosphorylation of 4E-BP1 and S6K1 was examined by incubating cells with varying concentrations of each amino acid (0.2, 0.5, 1, 5, or 10 mM). Phosphorylation of 4E-BP1 and S6K1 increased in proportion to the level of Leu supplementation (Figure 4A). A dose-dependent correlation was observed between the level of Arg supplementation and the phosphorylation of 4E-BP1 and S6K1 (Figure 4B). However, the extent of the increase in phosphorylation of 4E-BP1 and S6K1 in response to increasing concentration was less pronounced with Arg than with Leu.

We also examined the time course of 4E-BP1 and S6K1 phosphorylation after supplementation with 5 mM of Leu or Arg. Phosphorylation of 4E-BP1 and S6K1 in cells treated with Leu reached a maximum at 15 min and 30 min, respectively, then gradually decreased (Figure 4C). Although 4E-BP1 and S6K1 showed a similar time course of phosphorylation in cells treated with Arg, the extent of change was less pronounced than that observed in Leu-treated cells (Figure 4D).

Leu is known to act through an mTORC1-dependent pathway to stimulate the phosphorylation of 4E-BP1 and S6K1. We therefore attempted to confirm that Arg stimulates the phosphorylation of 4E-BP1 and S6K1 through the mTORC1 pathway. Treatment with rapamycin, a specific inhibitor of mTORC1, completely suppressed the phosphorylation of 4E-BP1 and S6K1 induced by both Arg and Leu (Figure 4E). These results indicate that, similar to Leu, Arg stimulates the phosphorylation of 4E-BP1 and S6K1 through the mTORC1 pathway.

### 3.4. Effect of Arg Metabolites on Translation Initiation in C2C12 Myotubes

**Figure 5:**
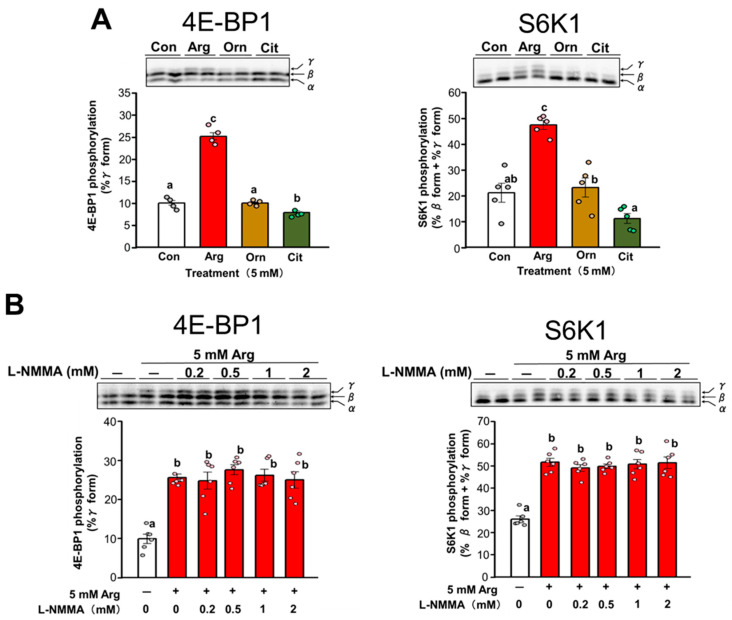
Effect of Arg metabolites on translation initiation in C2C12 myotubes. Differentiated C2C12 myotubes were starved of serum and amino acids for 4 h before being subjected to each experiment. Cell lysates were prepared and subjected to SDS-PAGE, and then the phosphorylation of 4E-BP1 and S6K1 was evaluated by immunoblot analysis. (**A**) Effect of Orn and Cit, key products of Arg metabolism, on the phosphorylation of 4E-BP1 and S6K1. Cells were treated with 5 mM Arg, Orn, or Cit for 15 min. (**B**) Effect of the NOS inhibitor L-NMMA on Arg-induced 4E-BP1 and S6K1 phosphorylation. Cells were pre-treated with 0–2 mM L-NMMA for 1 h and then incubated with 5 mM Arg for 15 min. Each value is the mean ± SE; *n* = 4–6. Values not sharing the same superscript letter differed significantly (*p* < 0.05) according to the Tukey-Kramer multiple comparison test.

Arg is metabolized to ornithine (Orn) and urea by the enzyme arginase. Arg is also metabolized to citrulline (Cit) and NO by the enzyme nitric oxide synthase (NOS) [32,33]. Orn and Cit are reportedly capable of stimulating translation initiation [34,35]. Therefore, the effect of Orn and Cit on the phosphorylation of 4E-BP1 and S6K1 was investigated in C2C12 myotubes. As shown in Figure 5A, neither Orn nor Cit had any effect on the phosphorylation of 4E-BP1 and S6K1. Furthermore, to investigate the possible involvement of NO in the Arg-induced stimulation of translation initiation, C2C12 myotubes were incubated with Arg in the presence or absence of various concentrations of the NOS inhibitor L-NMMA. L-NMMA did not attenuate the Arg-induced phosphorylation of 4E-BP1 and S6K1 (Figure 5B). These data suggest that Arg-induced stimulation of translation initiation is not dependent on the production of NO from Arg. Overall, these results indicate that Arg itself rather than its metabolites affects the phosphorylation of 4E-BP1 and S6K1, leading to an increase in translation initiation.

### 3.5. Involvement of the PI3K/AKT and MAPK Pathways in Arg-Induced Stimulation of Translation Initiation in C2C12 Myotubes

**Figure 6:**
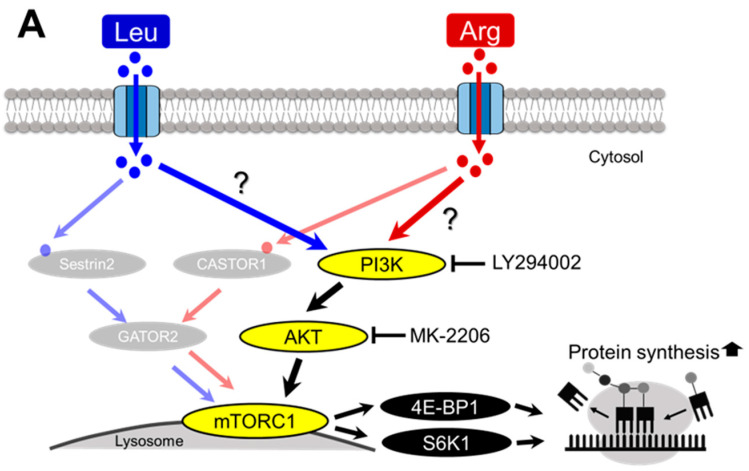

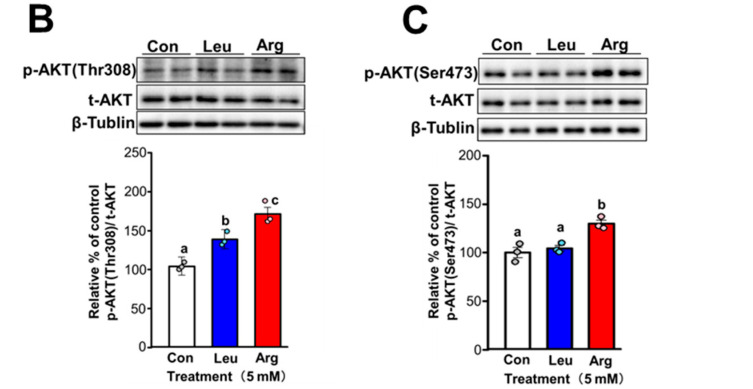

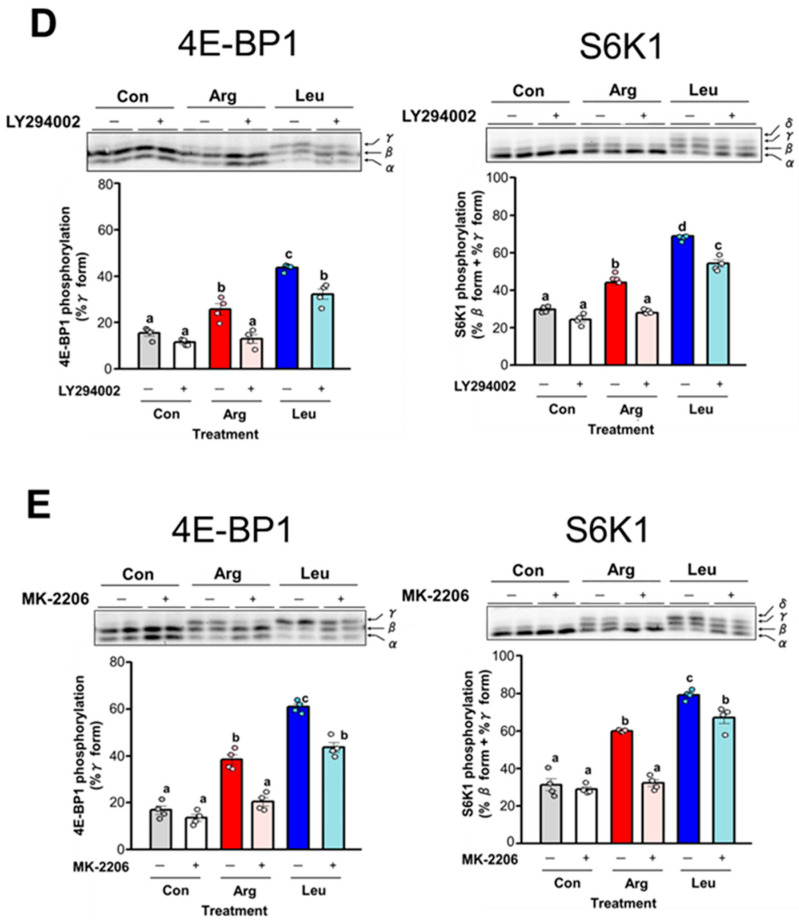
Involvement of the PI3K/AKT pathway in Arg- and Leu-induced stimulation of translation initiation in C2C12 myotubes. Differentiated C2C12 myotubes were starved of serum and amino acids for 4 h before being subjected to each experiment. Cell lysates were prepared and subjected to SDS-PAGE, and then the phosphorylation states of AKT, 4E-BP1, and S6K1 were evaluated by immunoblot analysis. (**A**) Schematic representation of a potential mechanism by which Arg or Leu stimulates translation initiation via the PI3K/AKT signaling pathway in skeletal muscle. (**B**,**C**) Effect of Arg or Leu on phosphorylation of AKT. Cells were incubated in amino acid–free medium supplemented with 5 mM Arg or Leu for 15 min, after which phosphorylation of AKT at Thr308 (**B**) and Ser473 (**C**) was analyzed. (**D**,**E**) Effect of PI3K/AKT pathway inhibition on Arg- or Leu-induced 4E-BP1 and S6K1 phosphorylation. Cells were pre-treated with or without 1 µM PI3K inhibitor LY294002 (**D**) or 0.5 µM AKT inhibitor MK-2206 (**E**) for 30 min and then incubated with 5 mM Arg or Leu for 15 min. Each value is the mean ± SE; *n* = 3–4. Values not sharing the same superscript letter differed significantly (*p* < 0.05) according to the Tukey-Kramer multiple comparison test.

The PI3K/AKT and MAPK pathways are major signaling pathways that modulate translation initiation [36,37,38]. Therefore, we investigated whether Arg-dependent stimulation of translation initiation occurs via these signaling pathways. We first investigated the involvement of the PI3K/AKT pathway in Arg-induced phosphorylation of 4E-BP1 and S6K1 in C2C12 myotubes (Figure 6A). Phosphorylated AKT was used as an indicator of PI3K/AKT activity. AKT is fully activated by phosphorylation of threonine 308 (Thr308) and serine 473 (Ser473) upon activation of PI3K signaling [39]. Thus, we examined AKT phosphorylation at these two sites after Leu or Arg treatment. Phosphorylation of AKT at Thr308 was significantly increased by both Leu and Arg treatments (Figure 6B). As shown in Figure 6C, Arg treatment significantly increased the phosphorylation of AKT at Ser473, whereas Leu treatment did not (Figure 6C). We next utilized the PI3K inhibitor LY294002 and AKT inhibitor MK-2206 to test the contribution of the PI3K/AKT pathway to the stimulation of Arg-induced 4E-BP1 and S6K1 phosphorylation. LY294002 abolished Arg-induced 4E-BP1 and S6K1 phosphorylation (Figure 6D) and also inhibited the Leu-induced phosphorylation of S6K1 and 4E-BP1, but not completely (Figure 6D). Similar to LY294002, MK-2206 completely inhibited Arg-induced 4E-BP1 and S6K1 phosphorylation and partially inhibited Leu-induced phosphorylation (Figure 6E). Taken together, these data indicate that PI3K/AKT is the main pathway by which Arg induces the phosphorylation of 4E-BP1 and S6K1. These data also suggested that activation of the PI3K/AKT pathway is involved in part in Leu-induced stimulation of 4E-BP1 and S6K1 phosphorylation.

**Figure 7:**
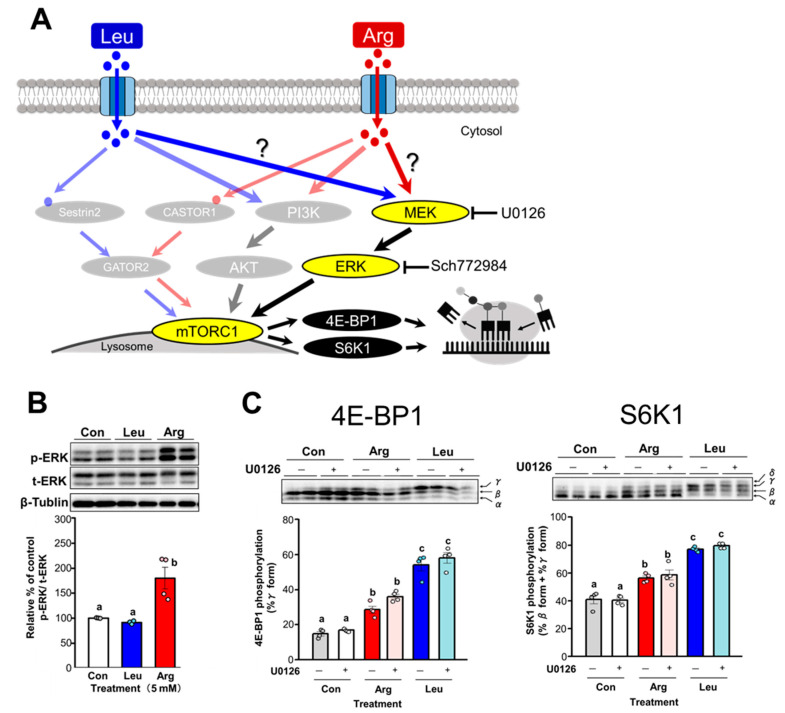

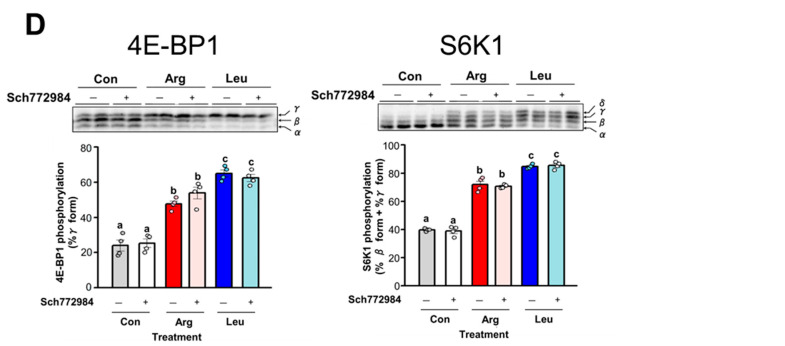
Involvement of the MAPK pathway in Arg- or Leu-induced stimulation of translation initiation in C2C12 myotubes. Differentiated C2C2 myotubes were starved of serum and amino acids for 4 h before being subjected to each experiment. Cell lysates were prepared and subjected to SDS-PAGE, and then the phosphorylation states of ERK, 4E-BP1, and S6K1 were evaluated by immunoblot analysis. (**A**) Schematic representation of the potential mechanism by which Arg or Leu stimulates translation initiation via the MAPK signaling pathway in skeletal muscle. (**B**) Effect of Arg or Leu on ERK phosphorylation. Cells were incubated in amino acid–free medium supplemented with 5 mM Arg or Leu for 15 min. (**C**,**D**) Effect of MAPK pathway inhibition on Arg- or Leu-induced 4E-BP1 and S6K1 phosphorylation. Cells were pre-treated with or without 0.5 µM MEK inhibitor U0126 (**C**) or 0.5 µM ERK inhibitor Sch772984 (**D**) for 30 min or 1 h, respectively, and then incubated with 5 mM Arg or Leu for 15 min. Each value is the mean ± SE; *n* = 3–4. Values not sharing the same superscript letter differed significantly (*p* < 0.05) according to the Tukey-Kramer multiple comparison test.

ERK phosphorylation levels were also measured as an indicator of MAPK pathway activity, as ERK is the final kinase in the MAPK pathway (Figure 7A). A significant increase in ERK phosphorylation was observed in Arg-treated cells but not Leu-treated cells (Figure 7B). The MEK inhibitor U0126 did not inhibit the phosphorylation of 4E-BP1 or S6K1 induced by either Arg or Leu treatment (Figure 7C). Similarly, the ERK inhibitor Sch772984 had no inhibitory effect on Arg- or Leu-induced phosphorylation of 4E-BP1 and S6K1 (Figure 7D). These results suggest that the MAPK pathway is activated by Arg treatment but not required for Arg-induced 4E-BP1 and S6K1 phosphorylation.

### 3.6. Involvement of GPRC6A in Arg-Induced Stimulation of Translation Initiation

**Figure 8:**
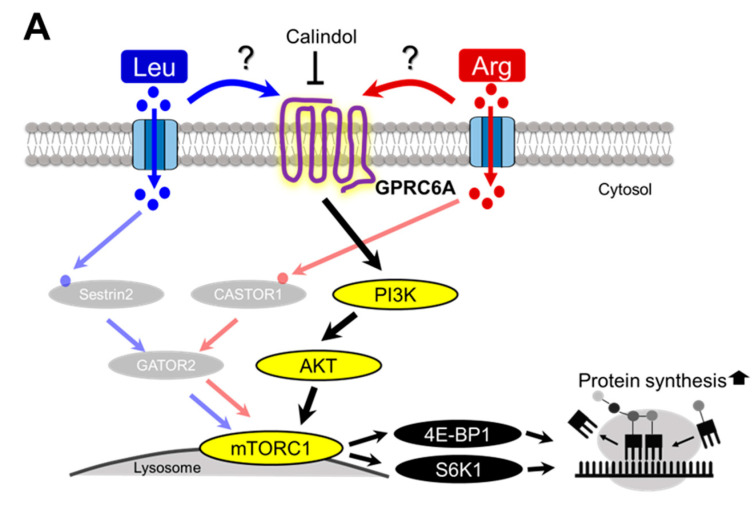

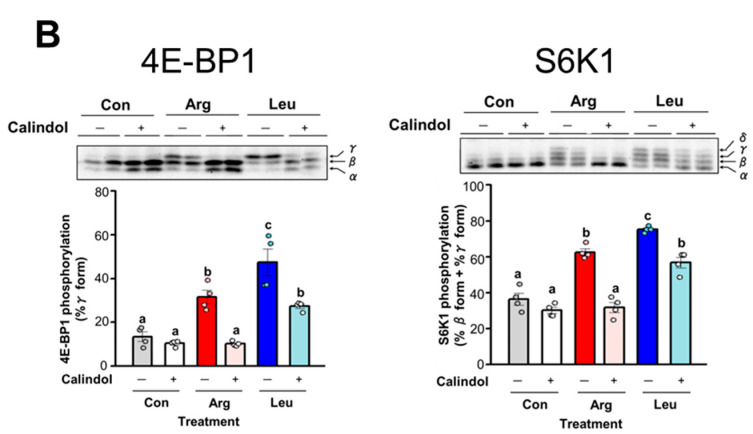
Contribution of GPRC6A in the signaling response to Arg or Leu stimulation in C2C12 myotubes. (**A**) Schematic representation of the GPRC6A signaling pathway through which Arg or Leu acts to stimulate translation initiation in skeletal muscle. (**B**) Effect of the GPRC6A inhibitor calindol on Arg- or Leu-induced phosphorylation of 4E-BP1 and S6K1. Differentiated C2C12 myotubes were starved of serum and amino acids for 3.5 h, pre-treated with or without 25 µM calindol for 30 min, and then incubated with 5 mM Arg or Leu for 15 min. Cell lysates were prepared and subjected to SDS-PAGE, and then the phosphorylation states of 4E-BP1 and S6K1 were evaluated by immunoblot analysis. Each value is the mean ± SE; *n* = 4. Values not sharing the same superscript letter differed significantly (*p* < 0.05) according to the Tukey-Kramer multiple comparison test.

The PI3K/AKT pathway is canonically activated by cell surface receptors, such as G protein–coupled receptors (GPCRs) and receptor tyrosine kinases, in response to extracellular stimuli such as growth factors and chemokines [40]. Arg-induced 4E-BP1 and S6K1 phosphorylation was abolished by treatment with the PI3K and AKT inhibitors, suggesting that Arg acts upstream of the PI3K/AKT pathway. GPRC6A (GPCR, Class C, group 6, subtype (A) is a receptor for amino acids such as lysine (Lys), Arg, and Orn that activates the PI3K/AKT pathway [41]. Therefore, we investigated whether GPRC6A is involved in Arg-induced stimulation of translation initiation (Figure 8A). Arg-induced 4E-BP1 and S6K1 phosphorylation was completely inhibited by treatment with the GPRC6A antagonist calindol (Figure 8B). By contrast, Leu-induced 4E-BP1 and S6K1 phosphorylation was suppressed by calindol treatment but still significantly increased compared with the control (Figure 8B).

## 4. Discussion

Arg plays a known role in muscle protein synthesis. Dietary Arg supplementation in milk-fed piglets has been shown to increase the phosphorylation of 4E-BP1, thereby enhancing assembly of the active eIF4G/eIF4E complex and upregulating protein synthesis in skeletal muscle [42]. Dietary Arg supplementation also reportedly upregulates muscle protein synthesis in lambs through the AKT/mTORC1 signaling pathway [43]. However, studies reported to date have primarily focused on the effect of chronic Arg administration on protein synthesis. Therefore, in the present study, we examined the effect of acute Arg administration on protein synthesis. We demonstrated for the first time that a single acute administration of Arg is sufficient to stimulate translation initiation in skeletal muscle of postabsorptive mice.

Arg is a recognized secretagogue of GH, which upregulates IGF-1 synthesis in the liver, thereby increasing its plasma concentration. IGF-1 is the main factor that stimulates an increase in skeletal muscle mass through upregulated protein synthesis and decreased proteolysis [44]. However, whether the effect of Arg on translation initiation is exerted through Arg itself or mediated via the GH/IGF-1 axis remains unclear. In the present study, oral administration of Arg significantly increased serum IGF-1 levels but did not affect the phosphorylation of S6K1. In contrast to oral administration, intraperitoneal injection of Arg significantly increased serum IGF-1 concentrations and induced significant phosphorylation of S6K1. These data demonstrate that the Arg-mediated stimulation of translation initiation is not solely attributable to the elevation in blood IGF-1 concentration caused by Arg administration. We therefore hypothesized that Arg stimulates translation initiation through a mechanism independent of IGF-1. Although there was no difference in serum IGF-1 levels between oral and intraperitoneal administration of Arg, serum Arg levels were more than twice as high following intraperitoneal administration compared with oral administration. Previous reports have shown that substantial amounts of orally administered Arg do not enter the systemic circulation in adults, as 40% of dietary Arg is degraded in the small intestine through first-pass metabolism [45]. In the present study, the serum concentration of Orn, a metabolite of Arg, increased 6-fold in Arg-treated mice compared with control mice, whereas the serum concentration of Arg increased by only 2-fold (Table 1). These results suggest that orally administered Arg is rapidly metabolized during intestinal transit. Therefore, it can be postulated that S6K1 phosphorylation following oral administration of Arg did not increase due to an insufficient increase in serum Arg concentration relative to that observed following intraperitoneal administration. Collectively, the results of in vivo analyses support the idea that Arg can exert a direct effect on the initiation of mRNA translation.

Oral administration of Arg had no observable effect on the phosphorylation state of S6K1, but intraperitoneal injection of Arg significantly increased S6K1 phosphorylation. This phenomenon can be attributed to the fact that intraperitoneal administration can lead to elevated serum Arg concentrations that are not achievable through oral administration. Therefore, the effect of Arg would be limited in a physiologically healthy animal, and even large doses of oral supplements may be insufficient. Several strategies are known to efficiently increase circulating Arg levels through oral Arg intake. For example, combined administration of Arg and Cit increases Arg concentration due to amino acid synergies [46]. In addition, in a number of species, including humans, rats, and pigs, endogenous synthesis of Arg becomes insufficient during the period of rapid growth, resulting in an increased physiological demand for Arg [47,48,49]. Under such conditions, even an orally administered dose of Arg may exert a pronounced stimulatory effect on translation initiation. Furthermore, elevation of serum Arg levels observed after intraperitoneal administration of Arg falls within the range achievable by intravenous infusion, and is not excessively high [50,51]. Taken together, these findings indicate that, under specific conditions, Arg can stimulate translation initiation even at physiological doses, providing important evidence supporting its potential role.

mTORC1 activity is regulated by growth factors and amino acids that signal through distinct but integrated molecular pathways [52]. The regulation of mTORC1 activity by growth factors is mediated by the PI3K/AKT signaling pathway, leading to phosphorylation and inhibition of TSC2 by AKT and subsequent activation of Rheb, which activates mTORC1 through an as-yet unknown mechanism [53]. Amino acid–dependent mTORC1 activation requires four Rag family small GTPases (RagA, RagB, RagC, and RagD) [54]. In the presence of amino acids, RagA binds GTP and RagC binds GDP, and the resulting heterodimer recruits mTORC1 to the lysosomal surface [55], which in turn allows growth factor signaling to activate mTORC1. Sestrin1/2 is a cytosolic Leu sensor, whereas CASTOR1 is a cytosolic Arg sensor. Upon binding of Leu or Arg to sestrin1/2 and CASTOR1, these proteins dissociate from GATOR2, which releases their suppressive effect on GATOR2. GATOR2 suppresses GATOR1 (a GTPase-activating protein (GAP)), which consequently activates mTORC1 via GTP-bound RagA formation by GAP inactivation.

A recent study demonstrated that oral administration of Leu to fasted rats promotes the dissociation of sestrin1 from GATOR2 [56]. This finding suggests that sestrin1 mediates Leu-induced activation of mTORC1 in skeletal muscle. Although we did not examine the involvement of sestrin1/2 in this study, Leu is thought to activate mTORC1 in part through an amino acid–sensing pathway involving sestrin1/2. Leu activates mTORC1 not only through Leu-dependent signaling pathways but also via insulin-dependent signaling pathways, as Leu also promotes insulin secretion [57,58]. Insulin is known to stimulate mTORC1 signaling by activating the PI3K/AKT signaling pathway [59,60]. In the present study, using C2C12 myotubes, neither insulin nor serum was included in the assay medium in order to eliminate any effect of insulin. Leu-induced 4E-BP1 and S6K1 phosphorylation was blocked by inhibitors of PI3K and AKT, showing that Leu activates the PI3K/AKT pathway. These results are consistent with previous reports showing that PI3K [61] and AKT [62] inhibitors abolish Leu-induced 4E-BP1 and S6K1 phosphorylation. However, PI3K and AKT inhibitors only partially blocked Leu-induced 4E-BP1 and S6K1 phosphorylation. Therefore, Leu is thought to stimulate the phosphorylation of 4E-BP1 and S6K1 not only through the PI3K/AKT pathway but also other pathways, such as the amino acid–sensing pathway involving sestrin1/2. Although the precise mechanism by which Leu stimulates the PI3K/AKT signaling pathway is unclear, it appears to involve LRS functioning as a Leu sensor [13] and the class III PI3K Vps34 (also known as PIK3C3) [63,64].

CASTOR1 was identified through in vitro studies as a cytosolic Arg sensor for the mTORC1 pathway. However, to the best of our knowledge, there are no reports describing the involvement of CASTOR1 in Arg-induced mTORC1 activation in vivo. Our in vitro studies using C2C12 myotubes showed that inhibitors of PI3K, AKT, and mTORC1 markedly suppressed Arg-induced 4E-BP1 and S6K1 phosphorylation. These results therefore suggest that Arg-induced stimulation of translation initiation is mediated by the PI3K/AKT/mTORC1 signaling pathway. Studies using several different types of cell models have shown that Arg cooperates with growth factor signaling to further promote the dissociation of TSC2 from lysosomes and the activation of mTORC1 [65]. The PI3K/AKT signaling pathway is a critical upstream regulator of TSC2 activity. Therefore, Arg appears to regulate mTORC1 through the PI3K/AKT/TSC2/Rheb signaling axis in C2C12 myotubes. Taken together, these data suggest that Arg and Leu stimulate the phosphorylation of 4E-BP1 and S6K1 in skeletal muscle through partially distinct signaling pathways.

GPRC6A is a receptor for basic amino acids such as Lys, Arg, and Orn and expressed in multiple tissues and organs, including skeletal muscle, and it participates in a variety of physiological activities in cells [66]. A recent study using mouse mammary epithelial cells showed that GPRC6A functions as a bridge that mediates communication between extracellular Arg and the intracellular PI3K/AKT/mTORC1 signaling pathway [67]. In our study using C2C12 myotubes, Arg-induced 4E-BP1 and S6K1 phosphorylation was abolished by PI3K and AKT inhibitors; therefore, we speculated that Arg acts on upstream signaling molecules of the PI3K/AKT pathway, such as GPRC6A. As expected, Arg-induced 4E-BP1 and S6K1 phosphorylation was completely inhibited in cells treated with the GPRC6A antagonist calindol. These results suggest that Arg activates the PI3K/AKT pathway via GPRC6A, leading to the phosphorylation of S6K1 and 4E-BP1, which in turn promotes the synthesis of muscle proteins. Calindol treatment also inhibited Leu-induced 4E-BP1 and S6K1 phosphorylation, suggesting that Leu acts on GPRC6A. However, calindol treatment only partially blocked Leu-induced 4E-BP1 and S6K1 phosphorylation, suggesting other pathways are involved in stimulating the phosphorylation of S6K1 and 4E-BP1. To date, there are no reports indicating that GPRC6A senses Leu. Further studies are thus necessary to examine Leu sensing by GPRC6A and to determine whether GPRC6A mediates Leu-induced mTORC1 activation.

## 5. Conclusions

Overall, the findings of this study suggest that Arg has a direct effect on the initiation of mRNA translation via the GPRC6A/PI3K/AKT/mTORC1 signaling pathway, thereby stimulating protein synthesis in skeletal muscle. Furthermore, our results also suggest that Leu stimulates translation initiation through multiple pathways, including the GPRC6A/PI3K/AKT/mTORC1 signaling pathway.

## Figures and Tables

**Table 1:** Serum amino acid concentrations in mice administered amino acids.

Amino Acid Concentration in Serum (nmol/mL)	Oral Administration			Intraperitoneal Administration		
	Con	Leu	Arg	Con	Leu	Arg
**Leu**	195.2 ± 9.1 ^a^	518.7 ± 40.7 ^b^	188.6 ± 18.4 ^a^	230.8 ± 26.8 ^a^	890.7 ± 43.7 ^b^	276.1 ± 21.5 ^a^
**Arg**	65.2 ± 4.8 ^a^	66.5 ± 8.2 ^a^	132.5 ± 15.8 ^b^	77.6 ± 4.5 ^a^	82.9 ± 11.8 ^a^	312.9 ± 62.7 ^b^
**Orn**	40.4 ± 4.5 ^a^	45.0 ± 5.8 ^a^	232.1 ± 27.2 ^b^	36.1 ± 2.9 ^a^	54.4 ± 4.5 ^a^	710.0 ± 65.2 ^b^

Different letters indicate significant differences between groups (*p* < 0.05). Values represent means ± SEM, *n* = 5–7. Data for oral and intraperitoneal administration were analyzed separately, and statistical comparisons were conducted within each administration route.

## Data Availability

The original contributions presented in this study are included in the article. Further inquiries can be directed to the corresponding author.
